# The stem cell zoo for comparative studies of developmental tempo^☆^

**DOI:** 10.1016/j.gde.2023.102149

**Published:** 2024-02

**Authors:** Jorge Lázaro, Jaroslaw Sochacki, Miki Ebisuya

**Affiliations:** 1 European Molecular Biology Laboratory (EMBL) Barcelona, Dr. Aiguader 88, 08003 Barcelona, Spain; 2 Collaboration for joint PhD degree between EMBL and Heidelberg University, Faculty of Biosciences, Heidelberg, Germany; 3 Cluster of Excellence Physics of Life, TU Dresden, Arnoldstraße 18, 01307 Dresden, Germany

## Abstract

The rate of development is highly variable across animal species. However, the mechanisms regulating developmental tempo have remained elusive due to difficulties in performing direct interspecies comparisons. Here, we discuss how pluripotent stem cell-based models of development can be used to investigate cell- and tissue-autonomous temporal processes. These systems enable quantitative comparisons of different animal species under similar experimental conditions. Moreover, the constantly growing stem cell zoo collection allows the extension of developmental studies to a great number of unconventional species. We argue that the stem cell zoo constitutes a powerful platform to perform comparative studies of developmental tempo, as well as to study other forms of biological time control such as species-specific lifespan, heart rate, and circadian clocks.

## Highlights

• Pluripotent stem cells can be used to model cell-intrinsic developmental tempo.
• The stem cell zoo allows developmental studies of unconventional species.
• Expansion of the stem cell zoo enables further interspecies tempo comparisons

**Current Opinion in Genetics & Development** 2024, **84**:102149

This review comes from a themed issue on **Developmental mechanisms, patterning and evolution (2024): Developmental Timing**

Edited by **James Briscoe** and **Miki Ebisuya**

For complete overview of the section, please refer to the article collection, “Developmental mechanisms, patterning and evolution (2024): Developmental Timing”

https://doi.org/10.1016/j.gde.2023.102149

0959–437X/© 2023 The Authors. Published by Elsevier Ltd. This is an open access article under the CC BY license (http://creativecommons.org/licenses/by/4.0/).

## Introduction

Spatiotemporal coordination of morphogenetic events is necessary for the correct unfolding of embryo development. A tight regulation of the time parameter is therefore essential during embryogenesis for controlling the size and cellular composition of tissues [1]. Embryos from different animal species, despite using conserved molecular mechanisms, display differences in their developmental tempo [2]. What regulates the rate of development across species? How is the species-specific tempo encoded in the genome? How did this trait evolve? All these remain open questions in the field.

The similarity between vertebrate embryos during the phylotypic stage has offered researchers an attractive system to tackle these questions [3]. During embryogenesis, vertebrates manifest a canonical body plan, with embryos of similar size and shape undergoing identical organogenetic events. However, the rate of development during embryogenesis remains species-specific, with human, mouse, chicken, and zebrafish finishing their organogenesis in approximately 60, 15, 10, and 3 days, respectively 4, 5. Direct interspecies comparisons of developmental tempo have remained challenging due to the different growing temperatures and uterine environments of individual animals. Only recently, the emergence of stem cell-based models of development has offered an unprecedented opportunity to investigate the regulation of developmental time across species [6]. *In vitro* differentiation of pluripotent stem cells (PSCs) can be used to recapitulate cell- or tissue-autonomous developmental processes and study them under similar experimental conditions. The use of stem cells also opens up the possibility to expand the developmental studies to unconventional animal species with interesting phenotypes whose embryos would be hard to obtain due to practical or ethical reasons. Additionally, stem cell systems are very amenable to genetic modification and highly quantitative measurements, allowing a finer dissection of the molecular players underlying developmental processes. In this review, we discuss the recent efforts in understanding the regulation of developmental tempo across species using stem cell models and the future of these *in vitro* systems.

## Interspecies comparisons of developmental tempo using a zoo of stem cells

Among the various examples of time control observed in development, the cell-autonomous gene expression oscillations of the segmentation clock represent an ideal system for comparing developmental tempo across species. The segmentation clock is active in the cells of the presomitic mesoderm (PSM) and controls the sequential formation of body segments along the vertebrate body axis [7]. This involves the molecular oscillations of the hairy and enhancer of split (HES) family of genes with a species-specific periodicity. *In vitro* modeling of the segmentation clock through the differentiation of PSCs into PSM cells has allowed the study of its temporal control in up to seven mammalian species, including mouse, rabbit, cattle, pig, rhinoceros, human, and marmoset 8••, 9, 10, 11, 12, 13, 14. This revealed that the period of the segmentation clock scales with the speed of HES7 biochemical reactions (e.g. protein degradation and intron delay) but not with the animal body weight, suggesting a general mechanism of tempo regulation that is uncoupled from a potential allometric dependency 8••, 9. Alterations of the HES7 biochemical kinetics can also influence the dynamics of the segmentation clock *in vivo* 15, 16, 17. Moreover, the HES7 biochemical reaction speeds do not scale with the cellular metabolic rate across species. This indicates that energy output cannot solely explain the species-specific biochemical kinetics [8]. However, perturbations of metabolic pathways in human PSM cells, specifically changes in the balance between the oxidixed and reduced forms of nicotinamide adenine dinucleotide (NAD+/NADH ratio), can slow down or accelerate the segmentation clock through changes in the translation rates, highlighting the importance of metabolism in the control of developmental time [18]. Changes in the glycolytic state have also been linked with alterations in PSM differentiation and segmentation *in vivo* 19, 20, 21. How biochemical kinetics are established so precisely and scaled across species, as well as the role of metabolism in their regulation, remains to be determined. Future studies focusing on the genetic control of developmental tempo will help us understand how are these species-specific phenotypes determined at the cellular level.

Stem cell-based models have also been widely used as a system to study developmental timing of neurogenesis. The specification of neuronal subtypes displays a species-specific temporal progression that can be recapitulated *in vitro* [22]. For example, spinal cord motor neuron differentiation takes 3–4 days in mouse but 2 weeks in human [23]. Differentiation of PSCs into spinal cord motor neurons revealed that, similar to the segmentation clock, the 2.5-times temporal scaling between mouse and human could be explained by changes in the biochemical reaction rates across species [24]. Another example of interspecies differences in tempo can be found during corticogenesis [25]. Human cortical neurons derived from PSCs show a prolonged timing of maturation as compared with other species, even when transplanted into mouse brains. This points to a cell-intrinsic mechanism for these developmental programs 26, 27, 28, 29. Metabolism has been shown to be a key regulator of cortical neuron maturation timing, with stimulation of mitochondrial metabolism being able to accelerate human neuronal maturation [30]. Moreover, a recent study highlighted the role of epigenetic regulation in the control of mouse and human cortical neuron maturation [31]. It will be important to address how similar the regulatory principles controlling the timing of neurogenesis are across different tissues and timescales, days in the case of spinal cord development and months for corticogenesis. Interestingly, extrinsic factors have also been found to impact the timing of neuronal differentiation, as shown by the acceleration of human neurogenesis during interspecies codifferentiation of human and mouse PSCs [32]. Stem cell-derived organoids, with their three-dimensional structures comprising several cell types, can be a useful platform to address the role of extrinsic factors and cell–cell interactions on developmental tempo. Brain organoids generated from human and other primate PSCs have revealed interspecies differences in the composition and rate of neuronal development 33••, 34. Similarly, self-organization of optic cup structures in retinal organoids takes longer and forms bigger structures in human compared with mouse [35]. The use of organoid models will allow researchers to understand how the cell-autonomous developmental tempo can be integrated at a tissue level and regulate species-specific morphogenesis.

The delay in development caused by responses to environmental stress, known as diapause, has recently emerged as a potential system to investigate the regulation of developmental tempo. This physiological process allows animals to reversibly slow down and pause their developmental progression in order to optimize the timing of birth [36]. Diapause has been observed naturally in numerous species, and can be recapitulated *in vitro* using preimplantation embryos as well as stem cell models 37, 38, 39•. Inhibition of the mammalian target of rapamycin (mTOR) pathway can reversibly induce pausing of mouse blastocysts, human stem cell-based blastocyst models (i.e. blastoids), and human and mouse PSCs 39•, 40. Interestingly, the speed at which the PSCs transition into a diapause-like state and some of the pathways involved in the maintenance of dormancy are different in mouse and human [39]. The mechanisms used by different species to regulate the entry and exit of diapause will be of great interest to uncover the key players involved in the control of developmental tempo.

## Exploring biological time: expansion of the zoo and its clocks

Most studies of developmental tempo involving stem cell models have focused on comparing mouse and human. This is mostly because these two species show large phenotypic differences and their stem cells have a great number of resources available for culture and differentiation. However, the study of other unconventional species is critical to assess the generality of the findings, determine what constitutes a species-specific behavior, and obtain more information about the evolutionary history of these developmental traits. For example, recapitulating *in vitro* the segmentation clock of six mammalian species has allowed the establishment of a general scaling law between the segmentation clock period and the speed of biochemical reactions [8]. Similarly, comparisons of human and primate brain organoids have revealed differences in the duration of particular developmental states that could explain the size expansion observed in human brains [33].

Reprogramming of adult somatic cells into induced pluripotent stem cells (iPSCs) has further enhanced our capacity to perform interspecies comparisons. Similar to embryonic stem cells (ESCs), iPSCs can proliferate indefinitely and differentiate into the three germ layers [41]. However, unlike ESCs, iPSCs are not derived from embryonic tissue, constituting a more practical stem cell source for many animal species. Over the years, a great amount of iPSC lines have been derived from wild and farm animals, generating a zoo of stem cells across the globe [42]. In Supplementary Table 1, we made a list of animal species with available PSCs, including bat, rhinoceros, seal, opossum, as well as several primates. The unique development of some of these species will allow the study of particular aspects of developmental time. For example, marsupial models of development could be useful to investigate the emergence of tissue-specific changes in developmental tempo, known as heterochronies. Marsupial embryogenesis is characterized by the faster development of the upper body as compared with the lower body, enabling them to climb into the pouch after birth and complete development 43, 44. *In vitro* modeling of embryonic structures with different anterior–posterior identities could offer valuable insight into the molecular basis of these differences in developmental tempo. It is important to point out that most PSC lines to date have been derived from mammals. Despite the efforts in obtaining stable stem cells from other vertebrate species, a better understanding of the mechanisms that allow the generation and maintenance of mammalian PSCs is necessary to derive high-quality stem cells from birds, reptiles, amphibians, and fishes 45, 46. Some of the currently available nonmammalian PSCs can only be considered as partially reprogrammed. Moreover, quantitative studies of developmental kinetics in cells from ectothermic animals will require the normalization of the different temperature conditions.

Apart from extending our current investigations to other animals, the use of PSCs from unconventional species could enable the study of other forms of biological time. One example is species-specific lifespan. Species with a long lifespan tend to have larger size and longer gestational periods [47]. Interestingly, slower biochemical reaction rates such as protein turnover or transcription elongation speed have been associated with increased longevity 48, 49•. These findings are consistent with the role of biochemical kinetics as regulators of developmental tempo. Species-specific mechanisms for extended lifespan are also reported, including the uniquely high efficiency and accuracy in DNA repair of the long-lived bowhead whale [50]. This is in line with the observations that somatic mutation rates are inversely correlated with lifespan across species [51]. Moreover, a pan-mammalian epigenetic signature has been seen to connect the methylation level of several developmental genes with the species’ maximum lifespan, suggesting a relation between aging and development 52, 53. Most of these studies have been done using adult tissue samples, allowing researchers to harvest cells form unconventional species in a relatively simple way. However, adult cells are very limited in their capacity to proliferate, making it challenging to keep them in culture and perform experimental perturbations. Stem cell systems provide an unlimited source of material, amenable to genetic modifications and with the capacity to differentiate to various tissues. The use of stem cells from uniquely long- or short-lived species could allow researchers to address whether lifespan signatures are present during embryonic development, as well as how they are initially established and maintained.

Another parameter correlated with life expectancy is the heart rate. Among mammals, with the exception of the human species, there is an inverse correlation between heart rate and lifespan. Animals that live longer tend to have slower heart beats, with a remarkably constant number of heart beats per lifetime across animals [54]. Similarly, larger animals tend to have slower heart rates as compared with animals of smaller size [55]. The heart beat is initiated by the pacemaker cells located in the sinoatrial node, and its frequency can be modulated through various neurotransmitters [56]. However, how the species-specific basal heart rate is first established remains to be determined. The strong correlation with body weight suggests a connection between heart rate and the organismal metabolic rate as a biophysical imperative. Nevertheless, a cell-autonomous mechanism for establishing basal heart rates in pacemaker cells could also be possible. Recent protocols have allowed the differentiation of PSCs into cardiac pacemaker cells 57, 58, 59. The study of these cells will enable researchers to investigate how species-specific heart rates are determined and what the influence of metabolism is on the beating frequency of cells and tissues.

PSCs could also help in the understanding of one of the most universal forms of biological time control found in nature: the circadian clock. This clock is an endogenous molecular feedback loop with a period of approximately 24 h that allows organisms to anticipate daily changes in the environment caused by earth’s rotation. The master circadian clock is located within the hypothalamus, but most cells in the body show circadian molecular oscillations [60]. The circadian clock appears during development, with PSCs having no apparent circadian oscillations [61]. Interestingly, the onset timing of the circadian clock appears to correlate with the rate of development, with rapidly developing species showing an earlier emergence of the clock [62]. *In vitro* models of PSC differentiation are already being used to understand the mechanisms regulating circadian clock appearance during development 61, 63. Furthermore, using PSCs from species living in polar environments — where regular photoperiods are largely absent — may provide valuable insights into the mechanisms allowing environment adaptation. Some of these species, such as arctic ground squirrel, have maintained their circadian clocks, while others, including reindeer, have lost their daily rhythmic activities 64, 65, 66.

Overall, the stem cell zoo offers a great platform to perform comparative studies of developmental processes. However, while PSC-derived models can recapitulate cell-intrinsic behaviors, they lack the ability to reproduce the complex environment and morphogenesis of the embryo. Xenotransplantation, species hybridization, or interspecies chimerism are useful approaches to address the importance of extrinsic factors in the establishment of species-specific behaviors *in vivo* 26, 67, 68. The combination of the stem cell zoo with embryological data is essential to avoid *in vitro* artifacts and have a comprehensive understanding of how developmental tempo emerges across species.

## Summary

**Figure 1:**
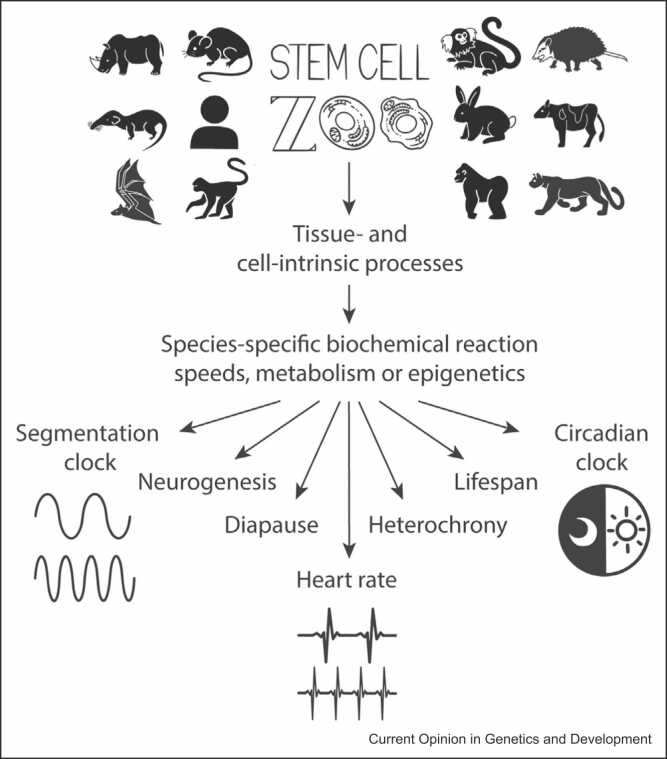
The stem cell zoo can be used to study temporal processes across species. PSCs from different animal species can be used to recapitulate tissue- and cell-autonomous mechanisms. Through changes in the speed of biochemical reactions, cellular metabolic rates, or epigenetics, PSC-derived models can effectively recapitulate species-specific differences across several biological processes.

The use of *in vitro* models of development has advanced our understanding of the rules governing developmental tempo. Stem cell differentiation allows us to compare different species under the same experimental conditions and learn about the mechanisms that make animal species unique at the cellular and tissue level (Figure 1). The addition of more species to the stem cell zoo will facilitate further interspecies comparisons at many biological levels.

## Funding

This work was supported by the 10.13039/100013060European Molecular Biology Laboratory (EMBL), the 10.13039/501100001659Deutsche Forschungsgemeinschaft (DFG, German Research Foundation) under Germany's Excellence Strategy — EXC 2068 — 390729961 — Cluster of Excellence Physics of Life of TU Dresden, the European Research Council (ERC) under the European Union’s Horizon 2020 research and innovation program (grant agreement No. 101002564) (to M.E.), and the 10.13039/501100001645Boehringer Ingelheim Fonds (BIF) PhD fellowship (to J.L.). M.E. is supported by the 10.13039/100005156Alexander von Humboldt Foundation in the framework of the Alexander von Humboldt Professorship endowed by the 10.13039/501100002347Federal Ministry of Education and Research10.13039/501100002347.

## CRediT authorship contribution statement

J.L. and M.E. conceived and wrote the paper. J.L. and J.S. assembled the list of animal species with available PSCs.

## Declaration of Competing Interest

The authors declare that they have no known competing financial interests or personal relationships that could have appeared to influence the work reported in this paper.

## Data Availability

No data were used for the research described in the article.

## Supplementary material

Supplementary material **Supplementary Table 1:** Animal species with available PSCs. Table containing a list of animal species with available PSCs. The type of PSCs is specified as embryonic stem cell (ESC) or induced pluripotent stem cell (iPSC). Note that the quality of the derived stem cell lines and the pluripotency tests performed can vary greatly between publications. For domestic animals and other common research models, not all available publications are included due to space limitations.

## References

[1] Ebisuya M., Briscoe J. (2018). What does time mean in development?. Dev.

[2] Xue L., Cai J.Y., Ma J., Huang Z., Guo M.X., Fu L.Z., Shi Y.B., Li W.X. (2013). Global expression profiling reveals genetic programs underlying the developmental divergence between mouse and human embryogenesis. BMC Genom.

[3] Duboule D. (1994). Temporal colinearity and the phylotypic progression: a basis for the stability of a vertebrate Bauplan and the evolution of morphologies through heterochrony. Development.

[4] Butler H., Juurlink B.H.J. (1987). An Atlas for Staging Mammalian and Chick Embryos.

[5] Kimmel C.B., Ballard W.W., Kimmel S.R., Ullmann B., Schilling T.F. (1995). Stages of embryonic development of the zebrafish. Dev Dyn.

[6] Rayon T., Briscoe J. (2021). **Cross-species comparisons and in vitro models to study tempo in development and homeostasis**. Interface Focus.

[7] Hubaud A., Pourquié O. (2014). Signalling dynamics in vertebrate segmentation. Nat Rev Mol Cell Biol.

[8••] Lázaro J., Costanzo M., Sanaki-Matsumiya M., Girardot C., Hayashi M., Hayashi K., Diecke S., Hildebrandt T.B., Lazzari G., Wu J. (2023). A stem cell zoo uncovers intracellular scaling of developmental tempo across mammals. Cell Stem Cell.

[9] Matsuda M., Hayashi H., Garcia-Ojalvo J., Yoshioka-Kobayashi K., Kageyama R., Yamanaka Y., Ikeya M., Toguchida J., Alev C., Ebisuya M. (2020). Species-specific segmentation clock periods are due to differential biochemical reaction speeds. Science.

[10] Diaz-Cuadros M., Wagner D.E., Budjan C., Hubaud A., Tarazona O.A., Donelly S., Michaut A., Al Tanoury Z., Yoshioka-Kobayashi K., Niino Y. (2020). In vitro characterization of the human segmentation clock. Nature.

[11] Chu L.F., Mamott D., Ni Z., Bacher R., Liu C., Swanson S., Kendziorski C., Stewart R., Thomson J.A. (2019). An in vitro human segmentation clock model derived from embryonic stem cells. Cell Rep.

[12] Matsumiya M., Tomita T., Yoshioka-Kobayashi K., Isomura A., Kageyama R. (2018). ES cell-derived presomitic mesoderm-like tissues for analysis of synchronized oscillations in the segmentation clock. Development.

[13] Matsuda M., Yamanaka Y., Uemura M., Osawa M., Saito M.K., Nagahashi A., Nishio M., Guo L., Ikegawa S., Sakurai S. (2020). Recapitulating the human segmentation clock with pluripotent stem cells. Nature.

[14] Conrad J.V., Meyer S., Ramesh P.S., Neira J.A., Rusteika M., Mamott D., Duffin B., Bautista M., Zhang J., Hiles E. (2023). Efficient derivation of transgene-free porcine induced pluripotent stem cells enables in vitro modeling of species-specific developmental timing. Stem Cell Rep.

[15] Harima Y., Takashima Y., Ueda Y., Ohtsuka T., Kageyama R. (2013). Accelerating the tempo of the segmentation clock by reducing the number of introns in the Hes7 gene. Cell Rep.

[16] Hirata H., Bessho Y., Kokubu H., Masamizu Y., Yamada S., Lewis J., Kageyama R. (2004). Instability of Hes7 protein is crucial for the somite segmentation clock. Nat Genet.

[17] Takashima Y., Ohtsuka T., González A., Miyachi H., Kageyama R. (2011). Intronic delay is essential for oscillatory expression in the segmentation clock. Proc Natl Acad Sci USA.

[18••] Diaz-Cuadros M., Miettinen T.P., Skinner O.S., Sheedy D., Díaz-García C.M., Gapon S., Hubaud A., Yellen G., Manalis S.R., Oldham W.M. (2023). Metabolic regulation of species-specific developmental rates. Nature.

[19] Bulusu V., Prior N., Snaebjornsson M.T., Kuehne A., Sonnen K.F., Kress J., Stein F., Schultz C., Sauer U., Aulehla A. (2017). Spatiotemporal analysis of a glycolytic activity gradient linked to mouse embryo mesoderm development. Dev Cell.

[20] Miyazawa H., Snaebjornsson M.T., Prior N., Kafkia E., Hammarén H.M., Tsuchida-Straeten N., Patil K.R., Beck M., Aulehla A. (2022). Glycolytic flux-signaling controls mouse embryo mesoderm development. Elife.

[21] Oginuma M., Moncuquet P., Xiong F., Karoly E., Chal J., Guevorkian K., Pourquié O. (2017). A gradient of glycolytic activity coordinates FGF and Wnt signaling during elongation of the body axis in amniote embryos. Dev Cell.

[22] Iwata R. (2022). Temporal differences of neurodevelopment processes between species. Neurosci Res.

[23] Davis-Dusenbery B.N., Williams L.A., Klim J.R., Eggan K. (2014). How to make spinal motor neurons. Dev.

[24] Rayon T., Stamataki D., Perez-Carrasco R., Garcia-Perez L., Barrington C., Melchionda M., Exelby K., Lazaro J., Tybulewicz V.L.J., Fisher E.M.C. (2020). Species-specific pace of development is associated with differences in protein stability. Science.

[25] Workman A.D., Charvet C.J., Clancy B., Darlington R.B., Finlay B.L. (2013). Modeling transformations of neurodevelopmental sequences across mammalian species. J Neurosci.

[26] Linaro D., Vermaercke B., Iwata R., Ramaswamy A., Libé-Philippot B., Boubakar L., Davis B.A., Wierda K., Davie K., Poovathingal S. (2019). Xenotransplanted human cortical neurons reveal species-specific development and functional integration into mouse visual circuits. Neuron.

[27] Marchetto M.C., Hrvoj-Mihic B., Kerman B.E., Yu D.X., Vadodaria K.C., Linker S.B., Narvaiza I., Santos R., Denli A.M., Mendes A.P.D. (2019). Species-specific maturation profiles of human, chimpanzee and bonobo neural cells. Elife.

[28] Otani T., Marchetto M.C., Gage F.H., Simons B.D., Livesey F.J. (2016). 2D and 3D stem cell models of primate cortical development identify species-specific differences in progenitor behavior contributing to brain size. Cell Stem Cell.

[29] Libé-Philippot B., Vanderhaeghen P. (2021). Cellular and molecular mechanisms linking human cortical development and evolution. Annu Rev Genet.

[30••] Iwata R., Casimir P., Erkol E., Boubakar L., Planque M., Gallego López I.M., Ditkowska M., Gaspariunaite V., Beckers S., Remans D. (2023). Mitochondria metabolism sets the species-specific tempo of neuronal development. Science.

[31•] Ciceri G., Cho H., Kshirsagar M., Baggiolini A., Aromolaran K.A., Walsh R.M., Goldstein P.A., Koche R.P., Leslie C.S., Studer L. (2022). An epigenetic barrier sets the timing of human neuronal maturation. bioRxiv.

[32] Brown J., Barry C., Schmitz M.T., Argus C., Bolin J.M., Schwartz M.P., van Aartsen A., Steill J., Swanson S., Stewart R. (2021). Interspecies chimeric conditions affect the developmental rate of human pluripotent stem cells. PLoS Comput Biol.

[33••] Benito-Kwiecinski S., Giandomenico S.L., Sutcliffe M., Riis E.S., Freire-Pritchett P., Kelava I., Wunderlich S., Martin U., Wray G.A., McDole K. (2021). An early cell shape transition drives evolutionary expansion of the human forebrain. Cell.

[34] Kanton S., Boyle M.J., He Z., Santel M., Weigert A., Sanchís-Calleja F., Guijarro P., Sidow L., Fleck J.S., Han D. (2019). Organoid single-cell genomic atlas uncovers human-specific features of brain development. Nature.

[35] Nakano T., Ando S., Takata N., Kawada M., Muguruma K., Sekiguchi K., Saito K., Yonemura S., Eiraku M., Sasai Y. (2012). Self-formation of optic cups and storable stratified neural retina from human ESCs. Cell Stem Cell.

[36] van der Weijden V.A., Bulut-Karslioglu A. (2021). Molecular regulation of paused pluripotency in early mammalian embryos and stem cells. Front Cell Dev Biol.

[37] Scognamiglio R., Cabezas-Wallscheid N., Thier M.C., Altamura S., Reyes A., Prendergast Á.M., Baumgärtner D., Carnevalli L.S., Atzberger A., Haas S. (2016). Myc depletion induces a pluripotent dormant state mimicking diapause. Cell.

[38] Hussein A.M., Wang Y., Mathieu J., Margaretha L., Song C., Jones D.C., Cavanaugh C., Miklas J.W., Mahen E., Showalter M.R. (2020). Metabolic control over mTOR-dependent diapause-like state. Dev Cell.

[39•] Iyer D.P., Weijden V.A., van der, Khoei H.H., McCarthy A., Rayon T., Simon C.S., Dunkel I., Wamaitha S.E., Elder K., Snell P. (2023). Delay of human early development via in vitro diapause. bioRxiv.

[40] Bulut-Karslioglu A., Biechele S., Jin H., MacRae T.A., Hejna M., Gertsenstein M., Song J.S., Ramalho-Santos M. (2016). Inhibition of mTOR induces a paused pluripotent state. Nature.

[41] Takahashi K., Tanabe K., Ohnuki M., Narita M., Ichisaka T., Tomoda K., Yamanaka S. (2007). Induction of pluripotent stem cells from adult human fibroblasts by defined factors. Cell.

[42] Friedrich Ben-Nun I., Montague S.C., Houck M.L., Tran H.T., Garitaonandia I., Leonardo T.R., Wang Y.C., Charter S.J., Laurent L.C., Ryder O.A. (2011). Induced pluripotent stem cells from highly endangered species. Nat Methods.

[43] Chew K.Y., Shaw G., Yu H., Pask A.J., Renfree M.B. (2014). Heterochrony in the regulation of the developing marsupial limb. Dev Dyn.

[44] Nunn C.L., Smith K.K. (1998). Statistical analyses of developmental sequences: the craniofacial region in marsupial and placental mammals. Am Nat.

[45] Rosselló R.A., Chen C.C., Dai R., Howard J.T., Hochgeschwender U., Jarvis E.D. (2013). Mammalian genes induce partially reprogrammed pluripotent stem cells in non-mammalian vertebrate and invertebrate species. Elife.

[46] Lu Y., West F.D., Jordan B.J., Mumaw J.L., Jordan E.T., Gallegos-Cardenas A., Beckstead R.B., Stice S.L. (2012). Avian-induced pluripotent stem cells derived using human reprogramming factors. Stem Cells Dev.

[47] Fushan A.A., Turanov A.A., Lee S.G., Kim E.B., Lobanov A.V., Yim S.H., Buffenstein R., Lee S.R., Chang K.T., Rhee H. (2015). Gene expression defines natural changes in mammalian lifespan. Aging Cell.

[48] Debès C., Papadakis A., Grönke S., Karalay Ö., Tain L.S., Mizi A., Nakamura S., Hahn O., Weigelt C., Josipovic N. (2023). Ageing-associated changes in transcriptional elongation influence longevity. Nature.

[49•] Swovick K., Firsanov D., Welle K.A., Hryhorenko J.R., Wise J.P., George C., Sformo T.L., Seluanov A., Gorbunova V., Ghaemmaghami S. (2021). Interspecies differences in proteome turnover kinetics are correlated with life spans and energetic demands. Mol Cell Proteom.

[50] Firsanov D., Zacher M., Tian X., Zhao Y., George J.C., Sformo T.L., Tombline G., Biashad S.A., Gilman A., Hamilton N. (2023). DNA repair and anti-cancer mechanisms in the longest-living mammal: the bowhead whale. bioRxiv.

[51] Cagan A., Baez-Ortega A., Brzozowska N., Abascal F., Coorens T.H.H., Sanders M.A., Lawson A.R.J., Harvey L.M.R., Bhosle S., Jones D. (2022). Somatic mutation rates scale with lifespan across mammals. Nature.

[52] Haghani A., Li C.Z., Robeck T.R., Zhang J., Lu A.T., Ablaeva J., Acosta-Rodríguez V.A., Adams D.M., Alagaili A.N., Almunia J. (2023). DNA methylation networks underlying mammalian traits. Science.

[53] Lu A.T., Fei Z., Haghani A., Robeck T.R., Zoller J.A., Li C.Z., Lowe R., Yan Q., Zhang J., Vu H. (2023). Universal DNA methylation age across mammalian tissues. Nat Aging.

[54] Levine H.J. (1997). Rest heart rate and life expectancy. J Am Coll Cardiol.

[55] Stahl W.R. (1967). Scaling of respiratory variables in mammals. J Appl Physiol.

[56] Kohajda Z., Loewe A., Tóth N., Varró A., Nagy N. (2020). The cardiac pacemaker story—fundamental role of the Na+/Ca2+ exchanger in spontaneous automaticity. Front Pharm.

[57] Wiesinger A., Li J., Fokkert L., Bakker P., Verkerk A.O., Christoffels V.M., Boink G.J.J., Devalla H.D. (2022). A single cell transcriptional roadmap of human pacemaker cell differentiation. Elife.

[58] Protze S.I., Liu J., Nussinovitch U., Ohana L., Backx P.H., Gepstein L., Keller G.M. (2016). Sinoatrial node cardiomyocytes derived from human pluripotent cells function as a biological pacemaker. Nat Biotechnol.

[59] Engel J.L., Zhang X., Lu D.R., Vila O.F., Arias V., Lee J., Hale C., Hsu Y.-H., Li C.-M., Wu R.S. (2023). Single cell multi-omics of an iPSC model of human sinoatrial node development reveals genetic determinants of heart rate and arrhythmia susceptibility. bioRxiv.

[60] Gachon F., Nagoshi E., Brown S.A., Ripperger J., Schibler U. (2004). The mammalian circadian timing system: from gene expression to physiology. Chromosoma.

[61] Yagita K., Horie K., Koinuma S., Nakamura W., Yamanaka I., Urasaki A., Shigeyoshi Y., Kawakami K., Shimada S., Takeda J. (2010). Development of the circadian oscillator during differentiation of mouse embryonic stem cells in vitro. Proc Natl Acad Sci USA.

[62] Dekens M.P.S., Whitmore D. (2008). Autonomous onset of the circadian clock in the zebrafish embryo. EMBO J.

[63] Umemura Y., Koike N., Tsuchiya Y., Watanabe H., Kondoh G., Kageyama R., Yagita K. (2022). Circadian key component CLOCK/BMAL1 interferes with segmentation clock in mouse embryonic organoids. Proc Natl Acad Sci USA.

[64] Van Oort B.E.H., Tyler N.J.C., Gerkema M.P., Folkow L., Blix A.S., Stokkan K.A. (2005). Circadian organization in reindeer. Nature.

[65] Lin Z., Chen L., Chen X., Zhong Y., Yang Y., Xia W., Liu C., Zhu W., Wang H., Yan B. (2019). Biological adaptations in the Arctic cervid, the reindeer (Rangifer tarandus). Science.

[66] Williams C.T., Barnes B.M., Yan L., Buck C.L. (2017). Entraining to the polar day: circadian rhythms in arctic ground squirrels. J Exp Biol.

[67] Seleit A., Brettell I., Fitzgerald T., Vibe C., Loosli F., Wittbrodt J., Naruse K., Birney E., Aulehla A. (2023). Modular control of time and space during vertebrate axis segmentation. bioRxiv.

[68] Wu J., Platero-Luengo A., Sakurai M., Sugawara A., Gil M.A., Yamauchi T., Suzuki K., Bogliotti Y.S., Cuello C., Morales Valencia M. (2017). Interspecies chimerism with mammalian pluripotent stem cells. Cell.

