# Supplementary material for: The stem cell zoo for comparative studies of developmental tempo
Source: Curr Opin Genet Dev. 2024 Feb;84:102149. doi: 10.1016/j.gde.2023.102149 (PMC10882223; doi:10.1016/j.gde.2023.102149)
Supplement: Supplementary file 1 — Supplementary material Supplementary Table 1: Animal species with available PSCs. Table containing a list of animal species with available PSCs. The type of PSCs is specified as embryonic stem cell (ESC) or induced pluripotent stem cell (iPSC). Note that the quality of the derived stem cell lines and the pluripotency tests performed can vary greatly between publications. For domestic animals and other common research models, not all available publications are included due to space limitations. [file mmc1.docx]

| Common name | Scientific name | Type of PSCs | References |
| --- | --- | --- | --- |
| **MAMMALS** |  |  |  |
| **PRIMATES** |  |  |  |
| Rhesus monkey | *Macaca mulatta* | iPSC, ESC | [1–8] |
| Olive baboon | *Papio anubis* | iPSC | [5,6] |
| Drill | *Mandrillus leucophaeus* | iPSC | [9] |
| Cynomolgus macaque | *Macaca fascicularis* | iPSC, ESC | [6,10–14] |
| Chimpanzee | *Pan troglodytes* | iPSC | [15–17] |
| Pig-Tailed macaque | *Macaca nemestrina* | iPSC | [17] |
| Bonobo | *Pan paniscus* | iPSC | [12,15] |
| Gorilla | *Gorilla gorilla* | iPSC | [12,18] |
| Orangutan | *Pongo abelii* | iPSC | [18,19] |
| Common Marmoset | *Callithrix jacchus* | iPSC, ESC | [20–28] |
| African green monkey | *Chlorocebus sabaeus* | iPSC | [29] |
| Human | *Homo sapiens* | iPSC, ESC | [30,31] |
| **RODENTIA** |  |  |  |
| Praire vole | *Microtus ochrogaster* | iPSC | [32,33] |
| Spiny rat | *Tokudaia osimensis* | iPSC | [34] |
| Naked mole rat | *Heterocephalus glaber* | iPSC | [35–37] |
| 13-lined ground squirrel | *Ictidomys tridecemlineatus* | iPSC | [38] |
| Rat | *Rattus norvegicus* | iPSC, ESC | [39–46] |
| Syrian hamster | *Mesocricetus auratus* | iPSC, ESC | [47,48] |
| Chinese hamster | *Cricetulus griseus* | iPSC | [49] |
| Mouse | *Mus musculus* | iPSC, ESC | [50–52] |
| **CHIROPTERA** |  |  |  |
| Little brown bat | *Myotis lucifugus* | iPSC | [53] |
| Wild greater horseshoe bat | *Rhinolophus ferrumequinum* | iPSC | [54] |
| Greater mouse-eared bat | *Myotis myotis* | iPSC | [54] |
| **CARNIVORA** |  |  |  |
| Snow leopard | *Panthera uncia* | iPSC | [55] |
| Bengal tiger | *Panthera tigris* | iPSC | [56] |
| Serval | *Leptailurus serval* | iPSC | [56] |
| Jaguar | *Panthera onca* | iPSC | [56] |
| American mink | *Neovison vison* | iPSC, ESC | [57,58] |
| Ferret | *Mustela putorius furo* | iPSC | [59,60] |
| Ringed seal | *Phoca hispida* | iPSC | [61] |
| Cat | *Felis catus* | iPSC, ESC | [62–65] |
| Dog | *Canis lupus familiaris* | iPSC, ESC | [66–74] |
| **ARTIODACTYLA** |  |  |  |
| Javan banteng | *Bos javanicus javanicus* | iPSC | [75] |
| Cow | *Bos taurus* | iPSC, ESC | [76–84] |
| Water buffalo | *Bubalus bubalis* | iPSC, ESC | [85–87] |
| Pig | *Sus scrofa domesticus* | iPSC, ESC | [88–96] |
| Goat | *Capra aegagrus hircus* | iPSC, ESC | [97–100] |
| Sheep | *Ovis aries* | iPSC, ESC | [101–107] |
| **PERISSODACTYLA** |  |  |  |
| Northern white rhinoceros | *Ceratotherium simum cottoni* | iPSC | [9,108,109] |
| Southern white rhinoceros | *Ceratotherium simum simum* | iPSC, ESC | [108,110] |
| Sumatran rhinoceros | *Dicerorhinus sumatrensis* | iPSC | [111] |
| Somali wild ass | *Equus africanus somaliensis* | iPSC | [75] |
| Horse | *Equus caballus* | iPSC, ESC | [112–118] |
| **LAGOMORPHA** |  |  |  |
| Rabbit | *Oryctolagus cuniculus* | iPSC, ESC | [119–127] |
| **DIDELPHIMORPHIA** |  |  |  |
| Gray short-tailed opossum | *Monodelphis domestica* | iPSC | [128] |
| **DASYUROMORPHIA** |  |  |  |
| Tasmanian devil | *Sarcophilus harrisii* | iPSC | [129] |
| **MONOTREMATA** |  |  |  |
| Platypus | *Ornithorhynchus anatinus* | iPSC | [130] |
| **AVIANS** |  |  |  |
| Chicken | *Gallus gallus* | iPSC, ESC | [131–138] |
| Quail | *Coturnix coturnix* | iPSC | [134,139] |
| Zebra finch | *Taeniopygia* | iPSC | [134] |
| Okinawa rail | *Hypotaenidia okinawae* | iPSC | [138] |
| Japanese ptarmigan | *Lagopus muta japonica* | iPSC | [138] |
| Blakiston’s fish owl | *Bubo blakistoni* | iPSC | [138] |
| Japanese golden eagle | *Aquila chrysaetos japonica* | iPSC | [138] |
| **FISHES** |  |  |  |
| Zebrafish | *Danio rerio* | iPSC, ESC | [134,140–144] |
| Medaka | *Oryzias latipes* | ESC | [145–148] |
| Sea bream | *Sparus aurata* | ESC | [149] |
| Red sea bream | *Chrysophrys major* | ESC | [150] |
| Sea perch | *Lateolabrax japonicus* | ESC | [151] |
| Atlantic cod | *Gadus morhua* | ESC | [152] |
| Asian sea bass | *Lates calcarifer* | ESC | [153] |
| Turbot | *Scophtalmus maximus* | ESC | [154] |
| Carp | *Cyprinus carpio haematopterus* | iPSC | [155] |

References

1. Liu H, Zhu F, Yong J, Zhang P, Hou P, Li H, Jiang W, Cai J, Liu M, Cui K, et al.: **Generation of Induced Pluripotent Stem Cells from Adult Rhesus Monkey Fibroblasts**. *Cell Stem Cell* 2008, **3**:587–590.

2. Fang R, Liu K, Zhao Y, Li H, Zhu D, Du Y, Xiang C, Li X, Liu H, Miao Z, et al.: **Generation of naive induced pluripotent stem cells from rhesus monkey fibroblasts**. *Cell Stem Cell* 2014, **15**:488–497.

3. Sosa E, Kim R, Rojas EJ, Hosohama L, Hennebold JD, Orwig KE, Clark AT: **An integration-free, virus-free rhesus macaque induced pluripotent stem cell line (riPSC90) from embryonic fibroblasts**. *Stem Cell Res* 2017, **21**:5–8.

4. Zhang X, Cao H, Bai S, Huo W, Ma Y: **Differentiation and characterization of rhesus monkey atrial and ventricular cardiomyocytes from induced pluripotent stem cells**. *Stem Cell Res* 2017, **20**:21–29.

5. Stauske M, Rodriguez Polo I, Haas W, Knorr DY, Borchert T, Streckfuss-Bömeke K, Dressel R, Bartels I, Tiburcy M, Zimmermann WH, et al.: **Non-Human Primate iPSC Generation, Cultivation, and Cardiac Differentiation under Chemically Defined Conditions**. *Cells* 2020, **9**.

6. Rodríguez-Polo I, Stauske M, Behr R: **Generation and Cultivation of Transgene-Free Macaque and Baboon iPSCs Under Chemically Defined Conditions**. *Methods Mol Biol* 2022, **2454**:697–716.

7. Pau KYF, Wolf DP: **Derivation and characterization of monkey embryonic stem cells**. *Reprod Biol Endocrinol* 2004, **2**:41.

8. Thomson JA, Kalishman J, Golos TG, Durning M, Harris CP, Becker RA, Hearn JP: **Isolation of a primate embryonic stem cell line**. *Proc Natl Acad Sci U S A* 1995, **92**:7844–7848.

9. Friedrich Ben-Nun I, Montague SC, Houck ML, Tran HT, Garitaonandia I, Leonardo TR, Wang YC, Charter SJ, Laurent LC, Ryder OA, et al.: **Induced pluripotent stem cells from highly endangered species**. *Nat Methods* 2011, **8**:829–831.

10. Shimozawa N: **Cynomolgus monkey induced pluripotent stem cells generated by using allogeneic genes**. *Methods Mol Biol* 2016, **1357**:173–182.

11. Coppiello G, Abizanda G, Aguado N, Iglesias E, Arellano-Viera E, Rodriguez-Madoz JR, Carvajal-Vergara X, Prosper F, Aranguren XL: **Generation of Macaca fascicularis iPS cell line ATCi-MF1 from adult skin fibroblasts using non-integrative Sendai viruses**. *Stem Cell Res* 2017, **21**:1–4.

12. Wunderlich S, Kircher M, Vieth B, Haase A, Merkert S, Beier J, Göhring G, Glage S, Schambach A, Curnow EC, et al.: **Primate iPS cells as tools for evolutionary analyses**. *Stem Cell Res* 2014, **12**:622–629.

13. Chen Y, Niu Y, Li Y, Ai Z, Kang Y, Shi H, Xiang Z, Yang Z, Tan T, Si W, et al.: **Generation of Cynomolgus Monkey Chimeric Fetuses using Embryonic Stem Cells**. *Cell Stem Cell* 2015, **17**:116–124.

14. Suemori H, Tada T, Torii R, Hosoi Y, Kobayashi K, Imahie H, Kondo Y, Iritani A, Nakatsuji N: **Establishment of embryonic stem cell lines from cynomolgus monkey blastocysts produced by IVF or ICSI**. *Dev Dyn* 2001, **222**:273–279.

15. Marchetto MCN, Narvaiza I, Denli AM, Benner C, Lazzarini TA, Nathanson JL, Paquola ACM, Desai KN, Herai RH, Weitzman MD, et al.: **Differential L1 regulation in pluripotent stem cells of humans and apes**. *Nature* 2013, **503**:525–529.

16. Romero IG, Pavlovic BJ, Hernando-Herraez I, Zhou X, Ward MC, Banovich NE, Kagan CL, Burnett JE, Huang CH, Mitrano A, et al.: **A panel of induced pluripotent stem cells from chimpanzees: A resource for comparative functional genomics**. *Elife* 2015, **4**:1–29.

17. Roodgar M, Suchy FP, Nguyen LH, Bajpai VK, Sinha R, Vilches-Moure JG, Van Bortle K, Bhadury J, Metwally A, Jiang L, et al.: **Chimpanzee and pig-tailed macaque iPSCs: Improved culture and generation of primate cross-species embryos**. *Cell Rep* 2022, **40**.

18. Geuder J, Wange LE, Janjic A, Radmer J, Janssen P, Bagnoli JW, Müller S, Kaul A, Ohnuki M, Enard W: **A non-invasive method to generate induced pluripotent stem cells from primate urine**. *Sci Rep* 2021, **11**:1–13.

19. Ramaswamy K, Yik WY, Wang XM, Oliphant EN, Lu W, Shibata D, Ryder OA, Hacia JG: **Derivation of induced pluripotent stem cells from orangutan skin fibroblasts Ecology**. *BMC Res Notes* 2015, **8**:1–9.

20. Yoshimatsu S, Nakajima M, Iguchi A, Sanosaka T, Sato T, Nakamura M, Nakajima R, Arai E, Ishikawa M, Imaizumi K, et al.: **Non-viral Induction of Transgene-free iPSCs from Somatic Fibroblasts of Multiple Mammalian Species**. *Stem Cell Reports* 2021, **16**:754–770.

21. Petkov S, Dressel R, Rodriguez-Polo I, Behr R: **Controlling the Switch from Neurogenesis to Pluripotency during Marmoset Monkey Somatic Cell Reprogramming with Self-Replicating mRNAs and Small Molecules**. *Cells* 2020, **9**.

22. Müller T, Fleischmann G, Eildermann K, Mätz-Rensing K, Horn PA, Sasaki E, Behr R: **A novel embryonic stem cell line derived from the common marmoset monkey (Callithrix jacchus) exhibiting germ cell-like characteristics**. *Hum Reprod* 2009, **24**:1359–1372.

23. Debowski K, Drummer C, Lentes J, Cors M, Dressel R, Lingner T, Salinas-Riester G, Fuchs S, Sasaki E, Behr R: **The transcriptomes of novel marmoset monkey embryonic stem cell lines reflect distinct genomic features**. *Sci Rep* 2016, **6**.

24. Nakajima M, Yoshimatsu S, Sato T, Nakamura M, Okahara J, Sasaki E, Shiozawa S, Okano H: **Establishment of induced pluripotent stem cells from common marmoset fibroblasts by RNA-based reprogramming**. *Biochem Biophys Res Commun* 2019, **515**:593–599.

25. Kishimoto K, Shimada A, Shinohara H, Takahashi T, Yamada Y, Higuchi Y, Yoneda N, Suemizu H, Kawai K, Kurotaki Y, et al.: **Establishment of novel common marmoset embryonic stem cell lines under various conditions**. *Stem Cell Res* 2021, **53**.

26. Wu Y, Zhang Y, Mishra A, Tardif SD, Hornsby PJ: **Generation of induced pluripotent stem cells from newborn marmoset skin fibroblasts**. *Stem Cell Res* 2010, **4**:180–188.

27. Thomson JA, Kalishman J, Golos TG, Durning M, Harris CP, Hearn JP: **Pluripotent cell lines derived from common marmoset (Callithrix jacchus) blastocysts**. *Biol Reprod* 1996, **55**:254–259.

28. Tomioka I, Maeda T, Shimada H, Kawai K, Okada Y, Igarashi H, Oiwa R, Iwasaki T, Aoki M, Kimura T, et al.: **Generating induced pluripotent stem cells from common marmoset (Callithrix jacchus) fetal liver cells using defined factors, including Lin28**. *Genes to Cells* 2010, **15**:959–969.

29. Chung YG, Seay M, Elsworth JD, Eugene Redmond D: **Generation of Pluripotent Stem Cells Using Somatic Cell Nuclear Transfer and Induced Pluripotent Somatic Cells from African Green Monkeys**. *Stem Cells Dev* 2020, **29**:1294–1307.

30. Thomson JA: **Embryonic stem cell lines derived from human blastocysts**. *Science* 1998, **282**:1145–1147.

31. Takahashi K, Tanabe K, Ohnuki M, Narita M, Ichisaka T, Tomoda K, Yamanaka S: **Induction of pluripotent stem cells from adult human fibroblasts by defined factors**. *Cell* 2007, **131**:861–872.

32. Katayama M, Hirayama T, Horie K, Kiyono T, Donai K, Takeda S, Nishimori K, Fukuda T: **Induced pluripotent stem cells with six reprogramming factors from prairie vole, which is an animal model for social behaviors**. *Cell Transplant* 2016, **25**:783–796.

33. Manoli DS, Subramanyam D, Carey C, Sudin E, van Westerhuyzen JA, Bales KL, Blelloch R, Shah NM: **Generation of induced pluripotent stem cells from the prairie vole**. *PLoS One* 2012, **7**:38119.

34. Honda A, Choijookhuu N, Izu H, Kawano Y, Inokuchi M, Honsho K, Lee AR, Nabekura H, Ohta H, Tsukiyama T, et al.: **Flexible adaptation of male germ cells from female iPSCs of endangered Tokudaia osimensis**. *Sci Adv* 2017, **3**.

35. Lee SG, Mikhalchenko AE, Yim SH, Lobanov A V., Park JK, Choi KH, Bronson RT, Lee CK, Park TJ, Gladyshev VN: **Naked Mole Rat Induced Pluripotent Stem Cells and Their Contribution to Interspecific Chimera**. *Stem Cell Reports* 2017, **9**:1706–1720.

36. Miyawaki S, Kawamura Y, Oiwa Y, Shimizu A, Hachiya T, Bono H, Koya I, Okada Y, Kimura T, Tsuchiya Y, et al.: **Tumour resistance in induced pluripotent stem cells derived from naked mole-rats**. *Nat Commun* 2016, **7**:1–9.

37. Tan L, Ke Z, Tombline G, Macoretta N, Hayes K, Tian X, Lv R, Ablaeva J, Gilbert M, Bhanu N V., et al.: **Naked Mole Rat Cells Have a Stable Epigenome that Resists iPSC Reprogramming**. *Stem Cell Reports* 2017, **9**:1721–1734.

38. Ou J, Ball JM, Luan Y, Zhao T, Miyagishima KJ, Xu Y, Zhou H, Chen J, Merriman DK, Xie Z, et al.: **iPSCs from a Hibernator Provide a Platform for Studying Cold Adaptation and Its Potential Medical Applications**. *Cell* 2018, **173**:851-863.e16.

39. Coppiello G, Abizanda G, Aguado N, Iglesias E, Iglesias-Garcia O, Lo Nigro A, Prosper F, Aranguren XL: **Generation of a Sprague-Dawley-GFP rat iPS cell line**. *Stem Cell Res* 2017, **21**:47–50.

40. Li W, Wei W, Zhu S, Zhu J, Shi Y, Lin T, Hao E, Hayek A, Deng H, Ding S: **Generation of Rat and Human Induced Pluripotent Stem Cells by Combining Genetic Reprogramming and Chemical Inhibitors (DOI:10.1016/j.stem.2008.11.014)**. *Cell Stem Cell* 2009, **4**:370.

41. Liao J, Cui C, Chen S, Ren J, Chen J, Gao Y, Li H, Jia N, Cheng L, Xiao H, et al.: **Generation of Induced Pluripotent Stem Cell Lines from Adult Rat Cells**. *Cell Stem Cell* 2009, **4**:11–15.

42. Takenaka-Ninagawa N, Kawabata Y, Watanabe S, Nagata K, Torihashi S: **Generation of rat-induced pluripotent stem cells from a new model of metabolic syndrome**. *PLoS One* 2014, **9**:104462.

43. Merkl C, Saalfrank A, Riesen N, Kühn R, Pertek A, Eser S, Hardt MS, Kind A, Saur D, Wurst W, et al.: **Efficient Generation of Rat Induced Pluripotent Stem Cells Using a Non-Viral Inducible Vector**. *PLoS One* 2013, **8**:e55170.

44. Li P, Tong C, Mehrian-Shai R, Jia L, Wu N, Yan Y, Maxson RE, Schulze EN, Song H, Hsieh CL, et al.: **Germline Competent Embryonic Stem Cells Derived from Rat Blastocysts**. *Cell* 2008, **135**:1299–1310.

45. Buehr M, Meek S, Blair K, Yang J, Ure J, Silva J, McLay R, Hall J, Ying QL, Smith A: **Capture of Authentic Embryonic Stem Cells from Rat Blastocysts**. *Cell* 2008, **135**:1287–1298.

46. Iannaccone PM, Taborn GU, Garton RL, Caplice MD, Brenin DR: **Pluripotent embryonic stem cells from the rat are capable of producing chimeras**. *Dev Biol* 1994, **163**:288–292.

47. Doetschman T, Williams P, Maeda N: **Establishment of hamster blastocyst-derived embryonic stem (ES) cells**. *Dev Biol* 1988, **127**:224–227.

48. Yoshimatsu S, Yamazaki A, Edamura K, Koushige Y, Shibuya H, Qian E, Sato T, Okahara J, Kishi N, Noce T, et al.: **Step-by-step protocols for non-viral derivation of transgene-free induced pluripotent stem cells from somatic fibroblasts of multiple mammalian species**. *Dev Growth Differ* 2022, **64**:325–341.

49. Pei H, Fu HY, Cho DS, Hu WS, Pei H, Cho DS, O’Brien TD, Dutton J, Hu WS, Pei H, et al.: **Generation of induced pluripotent stem cells from Chinese hamster embryonic fibroblasts**. *Stem Cell Res* 2017, **21**:132–136.

50. Martin GR: **Isolation of a pluripotent cell line from early mouse embryos cultured in medium conditioned by teratocarcinoma stem cells**. *Proc Natl Acad Sci U S A* 1981, **78**:7634–7638.

51. Evans MJ, Kaufman MH: **Establishment in culture of pluripotential cells from mouse embryos**. *Nature* 1981, **292**:154–156.

52. Takahashi K, Yamanaka S: **Induction of Pluripotent Stem Cells from Mouse Embryonic and Adult Fibroblast Cultures by Defined Factors**. *Cell* 2006, **126**:663–676.

53. Mo X, Li N, Wu S: **Generation and characterization of bat-induced pluripotent stem cells**. *Theriogenology* 2014, **82**:283.

54. Déjosez M, Marin A, Hughes GM, Morales AE, Godoy-Parejo C, Gray JL, Qin Y, Singh AA, Xu H, Juste J, et al.: **Bat pluripotent stem cells reveal unusual entanglement between host and viruses**. *Cell* 2023, **186**:957-974.e28.

55. Verma R, Holland MK, Temple-Smith P, Verma PJ: **Inducing pluripotency in somatic cells from the snow leopard (Panthera uncia), an endangered felid**. *Theriogenology* 2012, **77**:220-228.e2.

56. Verma R, Liu J, Holland MK, Temple-Smith P, Williamson M, Verma PJ: **Nanog is an essential factor for induction of pluripotency in somatic cells from endangered felids**. *Biores Open Access* 2013, **2**:72–76.

57. Menzorov AG, Matveeva NM, Markakis MN, Fishman VS, Christensen K, Khabarova AA, Pristyazhnyuk IE, Kizilova EA, Cirera S, Anistoroaei R, et al.: **Comparison of American mink embryonic stem and induced pluripotent stem cell transcriptomes**. *BMC Genomics* 2015, **16**:1–12.

58. Sukoyan MA, Vatolin SY, Golubitsa AN, Zhelezova AI, Semenova LA, Serov OL: **Embryonic stem cells derived from morulae, inner cell mass, and blastocysts of mink: Comparisons of their pluripotencies**. *Mol Reprod Dev* 1993, **36**:148–158.

59. Yoshimatsu S, Murakami R, Nakajima M, Sato T, Kawasaki H, Okano H: **Establishment of an induced pluripotent stem cell line from a female domestic ferret (Mustela putorius furo) with an X chromosome instability**. *Stem Cell Res* 2021, **53**:102385.

60. Gao J, Petraki S, Sun X, Brooks LA, Lynch TJ, Hsieh CL, Elteriefi R, Lorenzana Z, Punj V, Engelhardt JF, et al.: **Derivation of induced pluripotent stem cells from ferret somatic cells**. *Am J Physiol - Lung Cell Mol Physiol* 2020, **318**:L671–L683.

61. Beklemisheva VR, Belokopytova PS, Fishman VS, Menzorov AG: **Derivation of Ringed Seal (Phoca hispida) Induced Multipotent Stem Cells**. *Cell Reprogram* 2021, **23**:326–335.

62. Gómez MC, Serrano MA, Pope CE, Jenkins JA, Biancardi MN, López M, Dumas C, Galiguis J, Dresser BL: **Derivation of cat embryonic stem-like cells from in vitro-produced blastocysts on homologous and heterologous feeder cells**. *Theriogenology* 2010, **74**:498–515.

63. Yu X, Jin G, Yin X, Cho S, Jeon J, Lee S, Kong I: **Isolation and characterization of embryonic stem-like cells derived from in vivo-produced cat blastocysts**. *Mol Reprod Dev* 2008, **75**:1426–1432.

64. Dutton LC, Dudhia J, Guest DJ, Connolly DJ: **Inducing Pluripotency in the Domestic Cat (Felis catus)**. *Stem Cells Dev* 2019, **28**:1299–1309.

65. Zhou R, Comizzoli P, Keefer CL: **Endogenous pluripotent factor expression after reprogramming cat fetal fibroblasts using inducible transcription factors**. *Mol Reprod Dev* 2019, **86**:1671–1681.

66. Shimada H, Nakada A, Hashimoto Y, Shigeno K, Shionoya Y, Nakamura T: **Generation of canine-induced pluripotent stem cells by retroviral transduction and chemical inhibitors**. *Mol Reprod Dev* 2010, **77**:2.

67. Luo J, Suhr ST, Chang EA, Wang K, Ross PJ, Nelson LL, Venta PJ, Knott JG, Cibelli JB: **Generation of leukemia inhibitory factor and basic fibroblast growth factor-dependent induced pluripotent stem cells from canine adult somatic cells**. *Stem Cells Dev* 2011, **20**:1669–1678.

68. Whitworth DJ, Ovchinnikov DA, Wolvetang EJ: **Generation and characterization of LIF-dependent canine induced pluripotent stem cells from adult dermal fibroblasts**. *Stem Cells Dev* 2012, **21**:2288–2297.

69. Baird AEG, Barsby T, Guest DJ: **Derivation of Canine Induced Pluripotent Stem Cells**. *Reprod Domest Anim* 2015, **50**:669–676.

70. Lee AS, Xu D, Plews JR, Nguyen PK, Nag D, Lyons JK, Han L, Hu S, Lan F, Liu J, et al.: **Preclinical derivation and imaging of autologously transplanted canine induced pluripotent stem cells**. *J Biol Chem* 2011, **286**:32697–32704.

71. Koh S, Thomas R, Tsai S, Bischoff S, Lim JH, Breen M, Olby NJ, Piedrahita JA: **Growth requirements and chromosomal instability of induced pluripotent stem cells generated from adult canine fibroblasts**. *Stem Cells Dev* 2013, **22**:951–963.

72. Vaags AK, Rosic-Kablar S, Gartley CJ, Zheng YZ, Chesney A, Villagómez DAF, Kruth SA, Hough MR: **Derivation and Characterization of Canine Embryonic Stem Cell Lines with In Vitro and In Vivo Differentiation Potential**. *Stem Cells* 2009, **27**:329–340.

73. Hayes B, Fagerlie SR, Ramakrishnan A, Baran S, Harkey M, Graf L, Bar M, Bendoraite A, Tewari M, Torok-Storb B: **Derivation, Characterization, and In Vitro Differentiation of Canine Embryonic Stem Cells**. *Stem Cells* 2008, **26**:465–473.

74. Hatoya S, Torii R, Kondo Y, Okuno T, Kobayashi K, Wijewardana V, Kawate N, Tamada H, Sawada T, Kumagai D, et al.: **Isolation and characterization of embryonic stem-like cells from canine blastocysts**. *Mol Reprod Dev* 2006, **73**:298–305.

75. Ben-Nun IF, Montague SC, Houck ML, Ryder O, Loring JF: **Generation of induced pluripotent stem cells from mammalian endangered species**. *Methods Mol Biol* 2015, **1330**:101–109.

76. Sumer H, Liu J, Malaver-Ortega LF, Lim ML, Khodadadi K, Verma PJ: **NANOG is a key factor for induction of pluripotency in bovine adult fibroblasts**. *J Anim Sci* 2011, **89**:2708–2716.

77. Han X, Han J, Ding F, Cao S, Lim SS, Dai Y, Zhang R, Zhang Y, Lim B, Li N: **Generation of induced pluripotent stem cells from bovine embryonic fibroblast cells**. *Cell Res* 2011, **21**:1509–1512.

78. Cao H, Yang P, Pu Y, Sun X, Yin H, Zhang Y, Zhang Y, Li Y, Liu Y, Fang F, et al.: **Characterization of bovine induced pluripotent stem cells by lentiviral transduction of reprogramming factor fusion proteins**. *Int J Biol Sci* 2012, **8**:498–511.

79. Bogliotti YS, Wu J, Vilarino M, Okamura D, Soto DA, Zhong C, Sakurai M, Sampaio RV, Suzuki K, Izpisua Belmonte JC, et al.: **Efficient derivation of stable primed pluripotent embryonic stem cells from bovine blastocysts**. *Proc Natl Acad Sci U S A* 2018, **115**:2090–2095.

80. Saito S, Strelchenko N, Niemann H: **Bovine embryonic stem cell-like cell lines cultured over several passages**. *Roux’s Arch Dev Biol* 1992, **201**:134–141.

81. Talluri TR, Kumar D, Glage S, Garrels W, Ivics Z, Debowski K, Behr R, Niemann H, Kues WA: **Derivation and characterization of bovine induced pluripotent stem cells by transposon-mediated reprogramming**. *Cell Reprogram* 2015, **17**:131–140.

82. Zhao L, Gao X, Zheng Y, Wang Z, Zhao G, Ren J, Zhang J, Wu J, Wu B, Chen Y, et al.: **Establishment of bovine expanded potential stem cells**. *Proc Natl Acad Sci U S A* 2021, **118**:e2018505118.

83. Kawaguchi T, Tsukiyama T, Kimura K, Matsuyama S, Minami N, Yamada M, Imai H: **Generation of naïve bovine induced pluripotent stem cells using piggybac transposition of doxycycline-inducible transcription factors**. *PLoS One* 2015, **10**:e0135403.

84. Xiang J, Wang H, Zhang Y, Wang J, Liu F, Han X, Lu Z, Li C, Li Z, Gao Y, et al.: **LCDM medium supports the derivation of bovine extended pluripotent stem cells with embryonic and extraembryonic potency in bovine–mouse chimeras from iPSCs and bovine fetal fibroblasts**. *FEBS J* 2021, **288**:4394–4411.

85. Deng Y, Liu Q, Luo C, Chen S, Li X, Wang C, Liu Z, Lei X, Zhang H, Sun H, et al.: **Generation of induced pluripotent stem cells from buffalo (Bubalus bubalis) fetal fibroblasts with buffalo defined factors**. *Stem Cells Dev* 2012, **21**:2485–2494.

86. Verma V, Gautam SK, Singh B, Manik RS, Palta P, Singla SK, Goswami SL, Chauhan MS: **Isolation and characterization of embryonic stem cell-like cells from in vitro-produced buffalo (Bubalus bubalis) embryos**. *Mol Reprod Dev* 2007, **74**:520–529.

87. Anand T, Kumar D, Singh MK, Shah RA, Chauhan MS, Manik RS, Singla SK, Palta P: **Buffalo (Bubalus bubalis) Embryonic Stem Cell-Like Cells and Preimplantation Embryos Exhibit Comparable Expression of Pluripotency-Related Antigens**. *Reprod Domest Anim* 2011, **46**:50–58.

88. Wu Z, Chen J, Ren J, Bao L, Liao J, Cui C, Rao L, Li H, Gu Y, Dai H, et al.: **Generation of pig induced pluripotent stem cells with a drug-inducible system**. *J Mol Cell Biol* 2009, **1**:46–54.

89. Ezashi T, Telugu BPVL, Alexenko AP, Sachdev S, Sinha S, Roberts RM: **Derivation of induced pluripotent stem cells from pig somatic cells**. *Proc Natl Acad Sci U S A* 2009, **106**:10993–10998.

90. Chen LR, Shiue YL, Bertolini L, Medrano JF, BonDurant RH, Anderson GB: **Establishment of pluripotent cell lines from porcine preimplantation embryos**. *Theriogenology* 1999, **52**:195–212.

91. Notarianni E, Laurie S, Moor RM, Evans MJ: **Maintenance and differentiation in culture of pluripotential embryonic cell lines from pig blastocysts.** *J Reprod Fertil Suppl* 1990, **41**:51–56.

92. Esteban MA, Xu J, Yang J, Peng M, Qin D, Li W, Jiang Z, Chen J, Deng K, Zhong M, et al.: **Generation of induced pluripotent stem cell lines from Tibetan miniature pig**. *J Biol Chem* 2009, **284**:17634–17640.

93. Gao X, Nowak-Imialek M, Chen X, Chen D, Herrmann D, Ruan D, Chen ACH, Eckersley-Maslin MA, Ahmad S, Lee YL, et al.: **Establishment of porcine and human expanded potential stem cells**. *Nat Cell Biol* 2019, **21**:687–699.

94. Choi KH, Lee DK, Kim SW, Woo SH, Kim DY, Lee CK: **Chemically Defined Media Can Maintain Pig Pluripotency Network In Vitro**. *Stem Cell Reports* 2019, **13**:221–234.

95. West FD, Terlouw SL, Kwon DJ, Mumaw JL, Dhara SK, Hasneen K, Dobrinsky JR, Stice SL: **Porcine induced pluripotent stem cells produce chimeric offspring**. *Stem Cells Dev* 2010, **19**:1211–1220.

96. Conrad JV, Meyer S, Ramesh PS, Neira JA, Rusteika M, Mamott D, Duffin B, Bautista M, Zhang J, Hiles E, et al.: **Efficient derivation of transgene-free porcine induced pluripotent stem cells enables in vitro modeling of species-specific developmental timing**. *Stem cell reports* 2023, doi:10.1016/J.STEMCR.2023.10.009.

97. Ren J, Pak Y, He L, Qian L, Gu Y, Li H, Rao L, Liao J, Cui C, Xu X, et al.: **Generation of hircine-induced pluripotent stem cells by somatic cell reprogramming**. *Cell Res* 2011, **21**:849–853.

98. Kumar De A, Malakar D, Akshey YS, Jena MK, Dutta R: **Isolation and characterization of embryonic stem cell-like cells from in vitro produced goat (Capra hircus) embryos**. *Anim Biotechnol* 2011, **22**:181–196.

99. Behboodi E, Bondareva A, Begin I, Rao K, Neveu N, Pierson JT, Wylie C, Piero FD, Huang YJ, Zeng W, et al.: **Establishment of goat embryonic stem cells from in vivo produced blastocyst-stage embryos**. *Mol Reprod Dev* 2011, **78**:202–211.

100. Song H, Li H, Huang M, Xu D, Gu C, Wang Z, Dong F, Wang F: **Induced pluripotent stem cells from goat fibroblasts**. *Mol Reprod Dev* 2013, **80**:1009–1017.

101. Dattena M, Chessa B, Lacerenza D, Accardo C, Pilichi S, Mara L, Chessa F, Vincenti L, Cappai P: **Isolation, culture, and characterization of embryonic cell lines from vitrified sheep blastocysts**. *Mol Reprod Dev* 2006, **73**:31–39.

102. Zhao Y, Lin J, Wang L, Chen B, Zhou C, Chen T, Guo M, He S, Zhang N, Liu C, et al.: **Derivation and characterization of ovine embryonic stem-like cell lines in semi-defined medium without feeder cells**. *J Exp Zool Part A Ecol Genet Physiol* 2011, **315 A**:639–648.

103. Sartori C, Didomenico AI, Thomson AJ, Milne E, Lillico SG, Burdon TG, Whitelaw CBA: **Ovine-induced pluripotent stem cells can contribute to chimeric lambs**. *Cell Reprogram* 2012, **14**:8–19.

104. Li Y, Cang M, Lee AS, Zhang K, Liu D: **Reprogramming of sheep fibroblasts into pluripotency under a drug-inducible expression of mouse-derived defined factors**. *PLoS One* 2011, **6**:e15947.

105. Liu J, Balehosur D, Murray B, Kelly JM, Sumer H, Verma PJ: **Generation and characterization of reprogrammed sheep induced pluripotent stem cells**. *Theriogenology* 2012, **77**:338-346.e1.

106. Bao L, He L, Chen J, Wu Z, Liao J, Rao L, Ren J, Li H, Zhu H, Qian L, et al.: **Reprogramming of ovine adult fibroblasts to pluripotency via drug-inducible expression of defined factors**. *Cell Res* 2011, **21**:600–608.

107. German SD, Campbell KHS, Thornton E, Mclachlan G, Sweetman D, Alberio R: **Ovine induced pluripotent stem cells are resistant to reprogramming after nuclear transfer**. *Cell Reprogram* 2015, **17**:19–27.

108. Korody ML, Ford SM, Nguyen TD, Pivaroff CG, Valiente-Alandi I, Peterson SE, Ryder OA, Loring JF: **Rewinding Extinction in the Northern White Rhinoceros: Genetically Diverse Induced Pluripotent Stem Cell Bank for Genetic Rescue**. *Stem Cells Dev* 2021, **30**:177–189.

109. Zywitza V, Rusha E, Shaposhnikov D, Ruiz-Orera J, Telugu N, Rishko V, Hayashi M, Michel G, Wittler L, Stejskal J, et al.: **Naïve-like pluripotency to pave the way for saving the northern white rhinoceros from extinction**. *Sci Rep* 2022, **12**:1–18.

110. Hildebrandt TB, Hermes R, Colleoni S, Diecke S, Holtze S, Renfree MB, Stejskal J, Hayashi K, Drukker M, Loi P, et al.: **Embryos and embryonic stem cells from the white rhinoceros**. *Nat Commun* 2018, **9**:1–9.

111. Zywitza V, Frahm S, Krüger N, Weise A, Göritz F, Hermes R, Holtze S, Colleoni S, Galli C, Drukker M, et al.: **Induced pluripotent stem cells and cerebral organoids from the critically endangered Sumatran rhinoceros**. *iScience* 2022, **25**:105414.

112. Nagy K, Sung HK, Zhang P, Laflamme S, Vincent P, Agha-Mohammadi S, Woltjen K, Monetti C, Michael IP, Smith LC, et al.: **Induced Pluripotent Stem Cell Lines Derived from Equine Fibroblasts**. *Stem Cell Rev Reports* 2011, **7**:693–702.

113. Breton A, Sharma R, Diaz AC, Parham AG, Graham A, Neil C, Whitelaw CB, Milne E, Donadeu FX: **Derivation and characterization of induced pluripotent stem cells from equine fibroblasts**. *Stem Cells Dev* 2013, **22**:611–621.

114. Whitworth DJ, Ovchinnikov DA, Sun J, Fortuna PRJ, Wolvetang EJ: **Generation and characterization of leukemia inhibitory factor-dependent equine induced pluripotent stem cells from adult dermal fibroblasts**. *Stem Cells Dev* 2014, **23**:1515–1523.

115. Sharma R, Livesey MR, Wyllie DJA, Proudfoot C, Whitelaw CBA, Hay DC, Donadeu FX: **Generation of functional neurons from feeder-free, keratinocyte-derived equine induced pluripotent stem cells**. *Stem Cells Dev* 2014, **23**:1524–1534.

116. Khodadadi K, Sumer H, Pashaiasl M, Lim S, Williamson M, Verma PJ: **Induction of pluripotency in adult equine fibroblasts without c-MYC**. *Stem Cells Int* 2012, **2012**.

117. Saito S, Ugai H, Sawai K, Yamamoto Y, Minamihashi A, Kurosaka K, Kobayashi Y, Murata T, Obata Y, Yokoyama K: **Isolation of embryonic stem-like cells from equine blastocysts and their differentiation in vitro**. *FEBS Lett* 2002, **531**:389–396.

118. Li X, Zhou SG, Imreh MP, Ährlund-Richter L, Allen WR: **Horse embryonic stem cell lines from the proliferation of inner cell mass cells**. *Stem Cells Dev* 2006, **15**:523–531.

119. Honda A, Hirose M, Hatori M, Matoba S, Miyoshi H, Inoue K, Ogura A: **Generation of induced pluripotent stem cells in rabbits: Potential experimental models for human regenerative medicine**. *J Biol Chem* 2010, **285**:31362–31369.

120. Schoonjans L, Albright GM, Li JL, Collen D, Moreadith RW: **Pluripotential rabbit embryonic stem (ES) cells are capable of forming overt coat color chimeras following injection into blastocysts**. *Mol Reprod Dev* 1996, **45**:439–443.

121. Graves KH, Moreadith RW: **Derivation and characterization of putative pluripotential embryonic stem cells from preimplantation rabbit embryos**. *Mol Reprod Dev* 1993, **36**:424–433.

122. Fang ZF, Gai H, Huang YZ, Li SG, Chen XJ, Shi JJ, Wu L, Liu A, Xu P, Sheng HZ: **Rabbit embryonic stem cell lines derived from fertilized, parthenogenetic or somatic cell nuclear transfer embryos**. *Exp Cell Res* 2006, **312**:3669–3682.

123. Honda A, Hirose M, Inoue K, Ogonuki N, Miki H, Shimozawa N, Hatori M, Shimizu N, Murata T, Hirose M, et al.: **Stable embryonic stem cell lines in rabbits: Potential small animal models for human research**. *Reprod Biomed Online* 2008, **17**:706–715.

124. Intawicha P, Ou YW, Lo NW, Zhang SC, Chen YZ, Lin TA, Su HL, Guu HF, Chen MJ, Lee KH, et al.: **Characterization of embryonic stem cell lines derived from New Zealand white rabbit embryos**. *Cloning Stem Cells* 2009, **11**:27–37.

125. Afanassieff M, Tapponnier Y, Savatier P: **Generation of induced pluripotent stem cells in rabbits**. *Methods Mol Biol* 2016, **1357**:149–172.

126. Phakdeedindan P, Setthawong P, Tiptanavattana N, Rungarunlert S, Ingrungruanglert P, Israsena N, Techakumphu M, Tharasanit T: **Rabbit induced pluripotent stem cells retain capability of in vitro cardiac differentiation**. *Exp Anim* 2019, **68**:35–47.

127. Osteil P, Tapponnier Y, Markossian S, Godet M, Schmaltz-Panneau B, Jouneau L, Cabau C, Joly T, Blachère T, Gócza E, et al.: **Induced pluripotent stem cells derived from rabbits exhibit some characteristics of naïve pluripotency**. *Biol Open* 2013, **2**:613–628.

128. Kumar S, De Leon EM, Granados J, Whitworth DJ, VandeBerg JL: **Monodelphis domestica Induced Pluripotent Stem Cells Reveal Metatherian Pluripotency Architecture**. *Int J Mol Sci* 2022, **23**.

129. Weeratunga P, Shahsavari A, Ovchinnikov DA, Wolvetang EJ, Whitworth DJ: **Induced Pluripotent Stem Cells from a Marsupial, the Tasmanian Devil (Sarcophilus harrisii): Insight into the Evolution of Mammalian Pluripotency**. *Stem Cells Dev* 2018, **27**:112–122.

130. Whitworth DJ, Limnios IJ, Gauthier ME, Weeratunga P, Ovchinnikov DA, Baillie G, Grimmond SM, Graves JAM, Wolvetang EJ: **Platypus Induced Pluripotent Stem Cells: The Unique Pluripotency Signature of a Monotreme**. *Stem Cells Dev* 2019, **28**:151–164.

131. Yu P, Lu Y, Jordan BJ, Liu Y, Yang JY, Hutcheson JM, Ethridge CL, Mumaw JL, Kinder HA, Beckstead RB, et al.: **Nonviral minicircle generation of induced pluripotent stem cells compatible with production of chimeric chickens**. *Cell Reprogram* 2014, **16**:366–378.

132. Katayama M, Hirayama T, Tani T, Nishimori K, Onuma M, Fukuda T: **Chick derived induced pluripotent stem cells by the poly-cistronic transposon with enhanced transcriptional activity**. *J Cell Physiol* 2018, **233**:990–1004.

133. Fuet A, Montillet G, Jean C, Aubel P, Kress C, Rival-Gervier S, Pain B: **NANOG Is Required for the Long-Term Establishment of Avian Somatic Reprogrammed Cells**. *Stem Cell Reports* 2018, **11**:1272–1286.

134. Rosselló RA, Chen CC, Dai R, Howard JT, Hochgeschwender U, Jarvis ED: **Mammalian genes induce partially reprogrammed pluripotent stem cells in non-mammalian vertebrate and invertebrate species**. *Elife* 2013, **2**:e00036.

135. Pain B, Clark ME, Shen M, Nakazawa H, Sakurai M, Samarut J, Etches RJ: **Long-term in vitro culture and characterisation of avian embryonic stem cells with multiple morphogenetic potentialities**. *Development* 1996, **122**:2339–2348.

136. Boast S, Stern CD: **Simple methods for generating neural, bone and endodermal cell types from chick embryonic stem cells**. *Stem Cell Res* 2013, **10**:20–28.

137. Van De Lavoir MC, Mather-Love C, Leighton P, Diamond JH, Heyer BS, Roberts R, Zhu L, Winters-Digiacinto P, Kerchner A, Gessaro T, et al.: **High-grade transgenic somatic chimeras from chicken embryonic stem cells**. *Mech Dev* 2006, **123**:31–41.

138. Katayama M, Fukuda T, Kaneko T, Nakagawa Y, Tajima A, Naito M, Ohmaki H, Endo D, Asano M, Nagamine T, et al.: **Induced pluripotent stem cells of endangered avian species**. *Commun Biol* 2022, **5**:1–20.

139. Lu Y, West FD, Jordan BJ, Mumaw JL, Jordan ET, Gallegos-Cardenas A, Beckstead RB, Stice SL: **Avian-induced pluripotent stem cells derived using human reprogramming factors**. *Stem Cells Dev* 2012, **21**:394–403.

140. Peng L, Zhou Y, Xu W, Jiang M, Li H, Long M, Liu W, Liu J, Zhao X, Xiao Y: **Generation of stable induced pluripotent stem-like cells from adult zebra fish fibroblasts**. *Int J Biol Sci* 2019, **15**:2340–2349.

141. Hong N, Schartl M, Hong Y: **Derivation of stable zebrafish ES-like cells in feeder-free culture**. *Cell Tissue Res* 2014, **357**:623–632.

142. Collodi P, Kamei Y, Sharps A, Weber D, Barnes D: **Fish embryo cell cultures for derivation of stem cells and transgenic chimeras.** *Mol Mar Biol Biotechnol* 1992, **1**:257–265.

143. Sun Le, Bradford CS, Ghosh C, Collodi P, Barnes DW: **ES-like cell cultures derived from early zebrafish embryos**. *Mol Mar Biol Biotechnol* 1995, **4**:193–199.

144. Ho SY, Goh CWP, Gan JY, Lee YS, Lam MKK, Hong N, Hong Y, Chan WK, Shu-Chien AC: **Derivation and long-term culture of an embryonic stem cell-like line from zebrafish blastomeres under feeder-free condition**. *Zebrafish* 2014, **11**:407–420.

145. Hong Y, Winkler C, Schartl M: **Pluripotency and differentiation of embryonic stem cell lines from the medakafish (Oryzias latipes)**. *Mech Dev* 1996, **60**:33–44.

146. Hong Y, Schartl M: **Isolation and differentiation of medaka embryonic stem cells.** *Methods Mol Biol* 2006, **329**:3–16.

147. Yi M, Hong N, Hong Y: **Derivation and characterization of haploid embryonic stem cell cultures in medaka fish**. *Nat Protoc* 2010, **5**:1418–1430.

148. Hong N, He BP, Schartl M, Hong Y: **Medaka embryonic stem cells are capable of generating entire organs and embryo-like miniatures**. *Stem Cells Dev* 2013, **22**:750–757.

149. Béjar J, Hong Y, Alvarez MC: **An ES-like cell line from the marine fish Sparus aurata: Characterization and chimaera production**. *Transgenic Res* 2002, **11**:279–289.

150. Chen SL, Ye HQ, Sha ZX, Hong Y: **Derivation of a pluripotent embryonic cell line from red sea bream blastulas**. *J Fish Biol* 2003, **63**:795–805.

151. Chen SL, Sha ZX, Ye HQ, Liu Y, Tian YS, Hong Y, Tang QS: **Pluripotency and chimera competence of an embryonic stem cell line from the sea perch (Lateolabrax japonicus)**. *Mar Biotechnol* 2007, **9**:82–91.

152. Holen E, Kausland A, Skjærven K: **Embryonic stem cells isolated from Atlantic cod (Gadus morhua) and the developmental expression of a stage-specific transcription factor ac-Pou2**. *Fish Physiol Biochem* 2010, **36**:1029–1039.

153. Parameswaran V, Shukla R, Bhonde R, Hameed ASS: **Development of a pluripotent ES-like cell line from Asian sea bass (Lates calcarifer) - An oviparous stem cell line mimicking viviparous ES cells**. *Mar Biotechnol* 2007, **9**:766–775.

154. Holen E, Hamre K: **Towards obtaining long term embryonic stem cell like cultures from a marine flatfish, Scophtalmus maximus**. *Fish Physiol Biochem* 2003, **29**:245–252.

155. Xu W, Li H, Peng L, Pu L, Xiang S, Li Y, Tao L, Liu W, Liu J, Xiao Y, et al.: **Fish Pluripotent Stem-Like Cell Line Induced by Small-Molecule Compounds From Caudal Fin and its Developmental Potentiality**. *Front Cell Dev Biol* 2022, **9**:817779.
